# Risk factors for joint replacement in knee osteoarthritis; a 15-year follow-up study

**DOI:** 10.1186/s12891-017-1871-z

**Published:** 2017-12-04

**Authors:** Flemming K. Nielsen, Niels Egund, Anette Jørgensen, Anne Grethe Jurik

**Affiliations:** 1 Department of Radiology, Aarhus University Hospital, Noerrebrogade 44, 8000 Aarhus, Denmark; 2 Department of Rheumatology, Aarhus University Hospital, Noerrebrogade 44, 8000 Aarhus, Denmark

**Keywords:** Knee osteoarthritis, Radiography, Magnetic resonance imaging, Bone marrow lesion, Synovitis, Joint space narrowing

## Abstract

**Background:**

To evaluate whether clinical, radiographic or MRI findings are associated with long term risk for total knee arthroplasty (TKA) in persons with knee osteoarthritis.

**Methods:**

We performed a follow-up analysis of 100 persons with knee osteoarthritis who participated in a clinical trial between 2000 and 2002. Clinical data as well as radiography and MRI of the inclusion knee were obtained in all participants. Data on TKA procedures were extracted from The Danish National Patient Register. Clinical, radiographic and MRI findings were analyzed for associations with subsequent TKA.

**Results:**

During a mean follow-up period of 15 years, 66% received a TKA in the included knee (target knee); 37% also received a TKA in the other knee. The degree of joint space narrowing was highly associated with subsequent TKA (adjusted odds ratio (OR) 5.0 (95% confidence interval (95% CI) 2.6 – 9.9)) as was a radiological sum score comprising joint space narrowing, osteophytes, subchondral sclerosis and cysts (adjusted OR 1.7 (95% CI 1.3 – 2.1)). MRI detected bone marrow lesions, synovitis and effusion were similarly associated with subsequent TKA with an adjusted OR of 2.3 (95% CI 1.3 – 4.0), 2.8 (95% CI 1.5 – 5.2) and 1.9 (95% CI 1.2 – 3.1), respectively. Increased body mass index (BMI) was not associated with subsequent TKA in the target knee but was associated with TKA in the other knee (OR 2.3 (95% CI 1.2 – 4.3).

**Conclusions:**

Radiographic findings including joint space narrowing and MRI detected bone marrow lesions, synovitis and effusion were all significantly associated with the long term risk of TKA in persons with knee osteoarthritis.

**Electronic supplementary material:**

The online version of this article (10.1186/s12891-017-1871-z) contains supplementary material, which is available to authorized users.

## Background

Worldwide, osteoarthritis (OA) is the most prevalent joint disease and strongly associated with aging and obesity. The knee is the joint most commonly affected by OA with an estimated prevalence of 15% in persons aged 56 to 84 years [1].

The most important risk factors for knee OA include obesity, previous knee injury, and family history of OA [2]. The impact of each factor is debated but obesity is believed to be the most important. Thus, a recent systematic review found that onset of OA knee pain in persons over 50 years of age was related to overweight or obesity in 25% of cases; only 5% were caused by previous knee injury [3].

The end stage of OA is characterized by severe pain, disability and joint deformity and total knee arthroplasty (TKA) is often the only effective treatment. A recent study by Weinstein et al. [4] estimated that approximately 52% of adults diagnosed with symptomatic knee OA in the US will undergo TKA. Thus, approximately half of the population with knee OA will not progress to end stage disease and TKA. This has led some to suggest that other factors may be involved in disease manifestation than in disease progression [2, 5].

Conventional radiography is the most commonly used modality for imaging of knee OA although the radiographic changes in knee OA are generally not well correlated with symptoms [6]. However, the radiographic features of OA have been shown to correlate with disease progression and TKA [7, 8], especially the degree of joint space narrowing (JSN) [9]. Magnetic resonance imaging (MRI) offers the ability to visualize all structures in and around the knee, including cartilage, subchondral bone and soft tissue. Specific pathologic findings on MRI, including bone marrow lesions (BMLs) and synovitis, are moderately correlated with pain and have been associated with risk of TKA in studies with short term follow-up [9–12] as well as with progression of JSN [13, 14].

The purpose of our study was to analyze whether clinical, radiographic and MRI findings, including BML and synovitis, correlate with the incidence of TKA during a mean follow-up period of 15 years.

## Methods

### Data sources

The study was partly based on data from the following Danish registers:

The Danish Civil Registration System, established in 1968, assigns a 10-digit personal identification number to all Danish citizens at birth or immigration. This unique civil registration number can be used across all Danish registers and provide highly reliable data at individual level.

The Danish National Patient Register, established in 1977, includes data on all hospital in-patient contacts. Since 1995, data on out-patient contacts has also been included. Both public and private hospitals are obliged to report discharge diagnoses to the register, which are coded according to the Danish version of the International Classification of Diseases (ICD-10).

### Participants

Participants were selected from a previous multi-center, randomized, placebo-controlled double-blind trial including 337 participants comparing five intra-articular injections of Hyalgan® and placebo, respectively [15]. All included participants fulfilled the American College of Rheumatology (ACR) criteria for primary OA [16], had a Lequesne Algofunctional Index score of 10 or more (maximum score 24), a normal C-reactive protein level and e-GFR ≥ 60. The participants entered the study between January 2000 and December 2002. Exclusion criteria were secondary OA, inflammatory joint disease, significant OA symptoms from the other knee, radiographic OA in more than one tibiofemoral compartment, radiographic attrition >5 mm or severe co-morbidity (e.g. cancer and poor general health). One hundred and two participants were enrolled at our institution, constituting the potential study population for the present study.

**Fig. 1:**
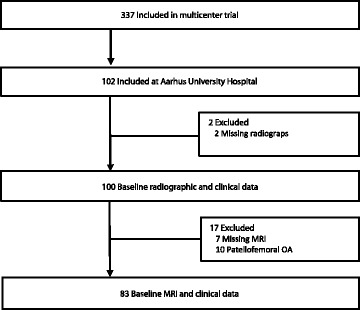
Diagram showing participant flow. MRI: magnetic resonance imaging; OA: osteoarthritis

Baseline radiographs were missing in two, leaving 100 participants with baseline radiographs and clinical data (Fig. 1). MRI was missing in seven participants with tibiofemoral OA, and 10 participants with only patellofemoral OA were excluded from the analyses of MR findings because the MRI protocol did not include an axial STIR sequence; thus, the MRI analyses were confined to 83 participants (Fig. 1).

The original study was approved by the Central Denmark Region Committee on Health Research Ethics and was carried out in accordance with the Declaration of Helsinki. Participants gave written informed consent prior to participation, including consent to publish study results and images. Prior to our follow-up study we contacted The Central Denmark Region Committees on Health Research Ethics who waived the need for ethical approval as no human biological material was involved. The study was approved by the Danish Data Protection Agency (1-16-02-126-16).

At inclusion, demographic variables (height and weight) were measured and all participants filled out a Western Ontario and McMaster Universities (WOMAC) questionnaire [17] consisting of the following items: Pain (5 items), stiffness (2 items) and physical function (17 items). Each score was made using a 100 mm Visual Analogue Scale (0 = no pain/stiffness/physical limitation, 100 = extreme pain/stiffness/physical limitation) with the following ranges: pain = 0-500, stiffness = 0-200, physical function = 0-1700, total score = 0-2400. All participants obtained radiography and MRI of the knee.

### Imaging

One leg weight-bearing radiographs in 30^0^ flexion of both knees were performed in three views: Postero-anterior and lateral views of the tibiofemoral, and axial view of the patellofemoral joint space. The initial lateral radiograph was used as guidance for securing optimal inclination of the lower leg for visualization of the tibiofemoral and patellofemoral joint spaces [18].

MR examinations were performed using a 1.5 Tesla system (Vision, Siemens, Erlangen, Germany) and a transmit receive four-channel knee coil. The examinations consisted of the following sequences: Sagittal STIR, repetition time (TR) = 5000 ms, echo time (TE) = 29 ms, inversion time = 150 ms, field of view (FOV) = 20 cm, slice thickness (ST) = 4.0 mm, interslice gap (IG) = 0.4 mm, matrix = 266 × 512 pixels, one excitation, and acquisition time (AT) 5.26 min; and sagittal and axial T1-weighted sequences. Gadodiamid (Omniscan, GE Healthcare, Norway) was injected at a peripheral intravenous site (0.1 mmol/kg with a maximum of 10 mmol) using a power injector followed by a saline flush. Sagittal and axial T1 fat suppressed contrast enhanced (T1 FS CE) sequences were performed using the following parameters: TR = 860 ms, TE = 20 ms, FOV = 16 cm, ST = 4.0 mm, IG = 0.8 mm, matrix = 512 × 512 pixels, one excitation, AT 7.23 min per sequence. All sagittal images were obtained perpendicular to the line connecting the dorsal aspect of the medial and lateral femoral condyle. Only the sagittal STIR and the sagittal and axial T1 FS CE images were analyzed in the present study.

### Follow-up data acquisition

Data on TKA procedures were extracted from the Danish National Patient Register, dividing the study population into a TKA and a non-TKA group.

### Image analysis

Radiographs and MR images were separated and anonymized prior to analysis, and readers were blinded to all study information, including outcome.

The radiographic definition of tibiofemoral (TF) OA was confined to visualization of joint space narrowing in either the medial or the lateral TF joint space. Joint space width of the medial TF articulation less than 3 mm was registered as joint space narrowing [19]. In addition, medial TF joint space larger than 3 mm but ≥1 mm reduced compared to the medial TF joint space of the other knee was considered joint space narrowing. Lateral TF joint space narrowing was registered when the joint space width was equal to or less than the medial TF joint space. Patellofemoral OA was registered when the medial and/or the lateral joint space width was less than 5 mm [20].

Radiographic grading was performed by NE and comprised assessment of JSN in the medial and lateral tibiofemoral and patellofemoral articulations according to a modified Ahlbäck grading [21, 22]. Grade 0 = normal joint spaces, grade 0.5 = < 50% JSN, grade 1.0 = > 50%, grade 1.5 = > 75% and grade 2.0 = 100% JSN; grade 3 = 100% JSN and < 5 mm bone attrition. Osteophytes (grades 0-3), subchondral sclerosis (0/1) and subchondral cysts (0/1) were also assessed. A radiographic sum score consisting of the scores for JSN, osteophytes, sclerosis and cysts was calculated.

BMLs at the tibiofemoral articulation were analyzed on the sagittal STIR images using a computer assisted segmentation (CAS) method described previously [23]. In brief, the method was based on pixel segmentation where a signal intensity threshold was calculated in the contralateral femoral condyle and tibial plateau which per definition were unaffected by radiographic OA (see exclusion criteria). The method has proven reliable in detecting even small BMLs compared to manual segmentation [23]. Because of relatively thick slices and interslice gap only three slices could reliably be measured without partial volume interference. The subchondral bone marrow in the affected femoral condyle and tibial plateau was outlined in all slices, excluding areas with partial volume from surrounding soft tissue. All pixels exceeding the threshold value, defined as the mean signal intensity in the radiographically unaffected compartment plus two standard deviations (SDs), were segmented by the CAS program yielding the BML size of the joint compartment relative to the entire bone marrow volume (relative BML).

All BML segmentations were performed twice by a radiological registrar (FKN) with at least three weeks between repeated measurements. Since inter-observer reliability has been high in a previous study [23], only intra-observer analyses were performed in the present study.

The relative BML size was ordered into categories using a 1 - 10 point scale (Grade 1 = 0 - 10% BML; grade 2 = >10 - 20% BML etc.).

Synovitis was assessed and graded according to the methods proposed by Guermazi et al. and Rhodes et al. [24, 25] on the axial and sagittal T1 FS CE images by NE and AGJ. The thickness of the enhanced synovium was measured at six sites, the medial and lateral parapatellar recesses at the level of the proximal half of the patella; the suprapatellar recess 1 – 1.5 cm cranial to the patella; at the ventral intercondylar area and dorsal surfaces of the medial and lateral femoral condyles. Grade 0 = no or patchy thin enhancement; grade 1 = even and continuous thickening of the synovial membrane <2 mm; grade 2 = thickening of the synovial membrane between 2 mm and 4 mm or nodular thickening between 2 mm and 4 mm; grade 3 = even thickening or nodular thickening between 4 mm and 8 mm and grade 4 = > 8 mm thickening. When the synovial space was separated by fluid, the thicknesses of the two adjacent synovial layers were summed. Sites with one synovial layer, e.g. the femoral condyle surfaces, were graded as follows: grade 1 = < 1 mm, grade 2 = 1 – 2 mm, grade 3 = 2 – 4 mm and grade 4 = > 4 mm. The synovitis score was summed over all six sites to one total score (maximum score 24). Effusion was measured in mm as the largest sagittal fluid distension of the suprapatellar recess on the midline sagittal T1 FS CE image. Intra- and inter-observer variation was based on 30 randomly chosen examinations.

Synovitis score was ordered into a five point ordinal scale (Grade 1 = 0 - <5; grade 2 = 5 - <10 etc.), and effusion score into an eight point ordinal scale (the maximum effusion score was 38) (Grade 1 = 0 - <5; grade 2 = 5 - <10 etc.).

### Statistical analyses

Data was analyzed using Stata 13.0 (StataCorp, College Station, TX, USA) and Analyse-it software (Analyse-it for Microsoft Excel (version 2.20) Ltd.; 2009).

Baseline characteristics were analyzed descriptively using means and SDs. Associations between baseline factors and any TKA were analyzed using univariate and multivariate logistic regression analyses. Multivariable logistic regression was performed using a single predictor adjusted for other variables (sex, age, body mass index). The extent of any significant association was expressed in odds ratio (OR) with 95% confidence interval (95% CI). Survival analysis comparing the cumulative incidence of TKA over time in relation to JSN, BML, synovitis and effusion was performed using Kaplan-Meier plots [24] and test for significance was performed using Cox proportional hazard ratio, adjusted for sex, age and BMI.

The association between baseline factors and unilateral vs. bilateral TKA was similarly analyzed using univariate and multivariable logistic regression.

Body mass index (BMI) was divided into four categories: Grade 1 (normal) = <25 kg/m^2^, Grade 2 (overweight) = 25 - <30 kg/m^2^, Grade 3 (obese) = 30 - <35 kg/m^2^ and Grade 4 (very obese) = ≥35 kg/m^2^.

In case of significant associations using logistic regression the scale used for measurement was ordered into categories using a maximum of 10 points.

Intra-observer reliability for BML measurements and intra- and inter-observer reliability for synovitis and effusion grading was assessed by Bland-Altman analyses, using plots, bias and 95% limits of agreement and by intraclass correlation coefficients.

## Results

Baseline characteristics for the 100 participants in the registry study are shown in Table 1. The mean follow-up time was 15.2 years (range 14.2 to 16.4 years). Ninety participants had tibiofemoral OA (80 medial, 10 lateral) and 10 had patellofemoral OA (six medial, four lateral). Baseline demographics of the 17 participants excluded from the MRI analyses due to lack of MRI or patellofemoral OA did not differ from the participants included in the MRI analyses.

**Table 1:** Baseline characteristics at inclusion and predictors of knee replacement, univariate and multivariate analyses

	Total	Knee replacement		Unadjusted		Adjusted^a^	
Variable	(*n* = 100)	Yes (*n* = 66)	No (*n* = 34)	OR (95% CI)	*p* value	OR (95% CI)	*p* value
Female, % (no.)	64% (64)	68% (45)	56% (19)	1.0 (0.9 – 1.0)	0.52		
Age (years), mean (SD)	62.0 (10.4)	61.3 (10.0)	63.4 (10.9)	1.0 (0.9 – 1.0)	0.34		
BMI grade, mean (SD)	2.2 (0.9)	2.2 (0.9)	2.0 (0.9)	1.2 (0.8 – 2.0)	0.41		
WOMAC, mean (SD):							
*- Pain*	184 (89)	196 (95)	161 (71)	1.0 (1.0 – 1.0)	0.07		
*- Stiffness*	81 (45)	87(46)	67 (40)	1.3 (1.01 – 1.6)^c^	0.04	1.3 (1.01 – 1.7)^c^	0.04
*- Function*	657 (319)	692 (332)	588 (281)	1.0 (1.0 – 1.0)	0.13		
*- Total*	922 (427)	976 (446)	817 (366)	1.0 (1.0 – 1.0)	0.08		
Hyalgan %, (no.)	48% (48)	50% (33)	44% (15)	1.3 (0.6 – 2.9)	0.58		
JSN grade, mean (SD)	1.7 (1.0)	2.0 (0.9)	1.0 (0.8)	3.6 (2.1 - 6.2)	<0.001	5.0 (2.6 - 9.9)	<0.001
Radiological sum score, mean (SD)	5.0 (2.6)	5.8 (2.3)	3.4 (2.5)	1.5 (1.3 - 1.9)	<0.001	1.7 (1.3 - 2.1)	<0.001
BML grade, mean (SD)^b^, ^d^	2.1 (1.4)	2.4 (1.5)	1.4 (0.9)	2.1 (1.2 – 3.7)	0.006	2.3 (1.3 – 4.0)	0.005
Synovitis grade, mean (SD)^b^, ^e^	2.6 (1.0)	2.9 (0.9)	2.0 (1.1)	2.7 (1.5 – 4.7)	0.001	2.8 (1.5 – 5.2)	0.001
Effusion grade, mean (SD)^b^, ^f^	2.4 (1.5)	2.7 (1.6)	1.7 (1.1)	1.9 (1.2 – 3.0)	0.005	1.9 (1.2 – 3.1)	0.006

*OR* odds ratio, *CI* confidence interval, *No*. number, *SD* standard deviation, *BMI* body mass index, *WOMAC* Western Ontario and McMaster Universities OA index, *JSN* joint space narrowing, *BML* bone marrow lesion

^a^Adjusted for sex, age and BMI; ^b^ Based on 83 participants with MRI scans and tibiofemoral osteoarthritis; ^c^ Logistic regression performed after ordering data into a ten point ordinal scale (Grade 1 = 0 – 10, grade 2 = 11 – 20 etc.); ^d^ Ordered into a ten point ordinal scale (Grade 1 = 0 - 10% BML, grade 2 = >10 - 20% BML etc.); ^e^Ordered into a five point ordinal scale (Grade 1 = 0 - <5; grade 2 = 5 - <10 etc.); ^f^ Ordered into an eight point ordinal scale (Grade 1 = 0 - < 5; grade 2 = 5 - <10 etc.) (maximum effusion score = 38)

A total of 66 participants had undergone TKA in the knee at follow-up; 37 had also obtained TKA in the other knee. There was no significant association between baseline demographics (sex, age, BMI) or Hyalgan treatment and the risk for TKA at follow-up (Table 1). There was a minor, though significant, association between stiffness item in WOMAC and TKA (adjusted OR 1.3 (95% CI 1.01 – 1.7)) but not with other WOMAC items.

**Fig. 2:**
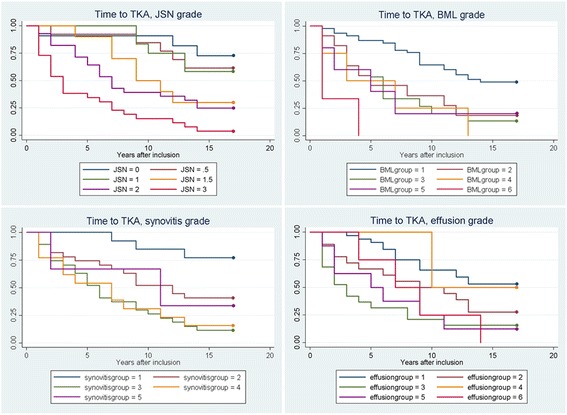
Kaplan-Meier plot showing the cumulative incidence of TKA in relation to different degrees of JSN, BML, synovitis and effusion**.** TKA: total knee arthroplasty; JSN: joint space narrowing; BML: bone marrow lesion

Significant associations between TKA and JSN (adjusted OR 5.0 (95% CI 2.6 – 9.9)), radiological sum score (adjusted OR 1.7 (95% CI 1.3 – 2.1)), BML (adjusted OR 2.3 (95% CI 1.3 – 4.0)), synovitis (adjusted OR 2.8 (95% CI 1.5 – 5.2)) and effusion (adjusted OR 1.9 (95% CI 1.2 – 3.1)) was observed (Table 1). The Kaplan-Meier time to event analysis illustrates the relation between increasing degrees of JSN, BML, synovitis and effusion and the risk of TKA (Fig. 2). Cox proportional hazard ratio showed a statistically significant hazard ratio for JSN, radiological sum score, BML, synovitis and effusion with the risk for TKA (Table 2).

**Table 2:** Cox proportional hazard ratios

	Unadjusted		Adjusted^a^	
Variable	Hazard ratio (95% CI)	*p* value	Hazard ratio (95% CI)	*p* value
JSN grade	2.2 (1.7 – 2.9)	<0.001	2.2 (1.7 – 3.0)	<0.001
Total radiological score	1.3 (1.1 – 1.4)	<0.001	1.3 (1.1 – 1.4)	<0.001
BML grade, ^b^	1.5 (1.2 – 1.7)	<0.001	1.5 (1.2 – 1.8)	<0.001
Synovitis grade, ^c^	1.5 (1.2 – 1.9)	0.001	1.5 (1.2 – 1.9)	0.001
Effusion grade, ^d^	1.3 (1.1 – 1.5)	0.002	1.3 (1.1 – 1.5)	0.001

*CI* confidence interval, *JSN* joint space narrowing, *BML* bone marrow lesion

^a^Adjusted for sex, age and BMI (body mass index); ^b^ Ordered into a ten point ordinal scale (Grade 1 = 0 - 10% BML, grade 2 = >10 - 20% BML etc.); ^c^ Ordered into a five point ordinal scale (Grade 1 = 0 - <5; grade 2 = 5 - <10 etc.); ^d^ Ordered into an eight point ordinal scale (Grade 1 = 0 - <5; grade 2 = 5 - <10 etc.) (maximum effusion score = 38)

The 66 participants with TKA at follow-up were compared in relation to unilateral versus bilateral TKA (Table 3); only BMI was associated with the risk of bilateral vs. unilateral TKA (OR 2.3 (95% CI 1.2 – 4.3)).

**Table 3:** Baseline characteristics and predictors of unilateral versus bilateral knee replacement, univariate and multivariate analyses

	Total	Knee replacement		Unadjusted		Adjusted^a^	
Variable	(n = 66)	Unilateral (*n* = 29)	Bilateral (*n* = 37)	OR (95% CI)	*p* value	OR (95% CI)	*p* value
Female, % (no.)	68% (45)	76% (22)	62% (23)	1.0 (0.9 – 1.0)	0.24		
Age (years), mean (SD)	61.3 (10.0)	62.9 (11.0)	59.9 (9.0)	0.5 (0.2 – 1.5)	0.23		
BMI, grade, mean (SD)	2.2 (0.9)	1.9 (0.9)	2.5 (0.9)	2.3 (1.2 – 4.3)	0.009		
WOMAC, mean (SD), baseline:							
*- Pain*	196 (95)	206 (91)	189 (98)	1.0 (1.0 – 1.0)	0.48	1.0 (1.0 – 1.0)	0.42
*- Stiffness*	87(46)	83 (47)	91 (44)	1.0 (1.0 – 1.0)	0.44	1.0 (1.0 – 1.0)	0.76
*- Function*	692 (332)	682 (320)	701 (340)	1.0 (1.0 – 1.0)	0.82	1.0 (1.0 – 1.0)	0.69
*- Total*	976 (446)	970 (420)	981 (465)	1.0 (1.0 – 1.0)	0.92	1.0 (1.0 – 1.0)	0.66
JSN grade, mean (SD)	2.0 (0.9)	1.9 (0.9)	2.1 (0.9)	1.3 (0.8 – 2.3)	0.30	2.2 (1.0 – 4.6)	0.05
Radiological sum score, mean (SD)	5.8 (2.3)	5.4 (2.4)	6.1 (2.2)	1.1 (0.9 – 1.4)	0.26	1.4 (1.0 – 1.9)	0.05
BML grade, mean (SD)^b^, ^c^	2.4 (1.6)	2.5 (1.7)	2.4 (1.5)	1.0 (0.7 – 1.3)	0.82	0.9 (0.6 – 1.3)	0.63
Synovitis grade, mean (SD)^b^, ^d^	2.9 (0.9)	2.8 (1.1)	2.9 (0.7)	1.2 (0.7 – 2.1)	0.61	1.1 (0.6 – 2.1)	0.78
Effusion grade, mean (SD)^b^, ^e^	2.7 (1.6)	2.4 (1.7)	3.0 (1.5)	1.3 (0.9 – 1.8)	0.20	1.2 (0.8 – 1.7)	0.39

*OR* odds ratio, *CI* confidence interval, *No*. number, *SD* standard deviation, *BMI* body mass index, *WOMAC* Western Ontario and McMaster Universities OA index, *JSN* joint space narrowing, *BML* bone marrow lesion

^a^Adjusted for sex, age and BMI; ^b^ Based on 56 participants with total knee arthroplasty, accessible MRI scan and tibiofemoral osteoarthritis; ^c^ Ordered into a ten point ordinal scale (Grade 1 = 0 - 10% BML, grade 2 = >10 - 20% BML etc.); ^d^ Ordered into a five point ordinal scale (Grade 1 = 0 - <5; grade 2 = 5 - <10 etc.); ^e^ Ordered into an eight point ordinal scale (Grade 1 = 0 - <5; grade 2 = 5 - <10 etc.) (maximum effusion score = 38)

The intra- and inter-observer agreement for all parameters were good (see Additional files 1, 2, 3 and 4). The intraclass correlation coefficients ranged between 0.88 – 0.99.

## Discussion

Our study showed that radiographic findings, specifically the degree of JSN, and MRI detected BML, synovitis and effusion were highly associated with TKA after 15 years of follow-up. Demographic characteristics such as sex, age or BMI were not correlated with the risk for TKA. There was, however, an association between higher BMI and the risk for bilateral TKA versus unilateral TKA.

The reason for the pronounced association between JSN and TKA could be explained by the surgical criteria for selecting patients with knee OA. According to the Danish national guidelines from 2012, the indications for TKA are primarily based on symptoms and objective findings [26]. It is, however, likely that the surgeon would generally not operate on a knee without radiographic signs of OA, especially JSN. Thus, a relative contra-indication for TKA is absence of or minimal radiologic changes [26]. No formalized Danish guidelines existed before 2012 but a comparison of preoperative knee scores from the Danish Registry of Knee Arthroplasty between 2005 and 2009 revealed no differences in disease severity [27]. Thus, there is no indication that TKA guidelines have changed between 2005 and 2009.

JSN has also previously been shown to be associated with knee OA progression. Oak et al. [28] found that baseline JSN was significantly correlated with worsening of knee osteoarthritis outcome score (KOOS) for pain, KOOS for symptoms and KOOS for quality of life at 4-year follow-up. In a study by Raynauld et al. [9], the degree of JSN was significantly associated with subsequent TKA during a follow-up period of six years.

BML, synovitis and effusion were all significantly associated with the risk of TKA and our results are thus in line with previous studies [9–12, 29]. However, while these studies had a relatively short follow-up period (one to six years) we showed that BML, synovitis and effusion were associated with long-term risk of TKA and that increasing degree of changes were associated with faster progression to TKA. MRI are not commonly used in the diagnosis of knee OA and the presence or absence of BML, synovitis or effusion are thus not likely to influence the surgeon’s decision to perform a TKA. Instead, BML and synovitis have been found associated with symptoms [30–32] and disease progression [33, 34], and our results imply that they play a central role in the disease process.

An association between baseline demographics (age, sex) and TKA at follow-up was not found. Although both age and sex are often highlighted as risk factors for knee OA, neither have consistently been associated with disease progression [7, 35]. Furthermore, we did not find any association between overall WOMAC score and subsequent TKA. The severity of symptoms and progression of knee OA have been associated in some studies [10, 36], often with a short follow-up. Our study showed that long-term risk of TKA was dependent on the amount of structural changes on radiographs and signs of synovial inflammation or BMLs on MR images.

No significant association was found between baseline BMI and TKA. However, among participants with TKA at follow-up, a higher BMI was associated with an increased risk of bilateral vs. unilateral TKA. Thus, our results suggest that a higher BMI does not increase the risk of TKA in knees with symptomatic OA. However, it seems that an increased BMI predisposes to development of OA in the opposite knee. Other studies have similarly reported that an elevated BMI predisposes to incident but not progression of knee OA [2, 5]. However, based on the amount of available data, we do not believe that we can draw any firm conclusions on this.

Our study has some limitations. The number of participants was limited compared to other longitudinal studies [10]. The absence of an axial STIR sequence implied that we were not able to analyze BML in participants with patellofemoral OA using computer-assisted segmentation. The signal intensity threshold was based on measurements in the contralateral joint compartment that was unaffected by radiographic OA. However, MRI changes, including BMLs, are frequently seen in radiographically normal joints and could affect BML calculations. Great care was therefore taken when outlining regions of interest for threshold calculations in the contralateral joint compartment to avoid areas with any signs of pathologic signal disturbances. Our BMI data was limited to baseline data only and we did not know whether BMI changed from baseline to the time of TKA. Furthermore, other pathologies associated with knee OA, e.g. meniscal or ligamentous damage, were not analyzed.

The strengths of our study were the use of a long follow-up period and a high reliability of registry TKA data. Our synovitis and effusion grading was based on contrast enhanced sequences which have been shown to be superior to fluid sensitive sequences.

## Conclusions

In conclusion, we found that radiographic changes associated with knee OA as well as MRI detected BMLs, synovitis and effusion were significantly associated with the risk of TKA in patients with knee OA. Demographic variables including BMI was not associated with TKA in knees already affected by OA. However, an increased BMI was seen in participants who developed knee OA requiring TKA in the opposite knee during follow-up.

## Additional files

Additional file 1: Table S1. Observer agreement, BML and synovitis grading, Bland-Altman analyses. (DOCX 12 kb)

Additional file 2: Table S2. Observer agreement, BML and synovitis grading, Intraclass correlation coefficients. (DOCX 12 kb)

Additional file 3: Figure S1. Inter-observer synovitis, Bland Altman plot. (DOCX 13 kb)

Additional file 4: Figure S2. Inter-observer effusion, Bland-Altman plot. (DOCX 13 kb)

## Abbreviations

BMI: Body mass index.
BML: Bone marrow lesion
CAS: Computer assisted segmentation
CI: Confidence interval
JSN: Joint space narrowing
MRI: Magnetic resonance imaging
OA: Osteoarthritis
OR: Odds ratio
SD: Standard deviation
TKA: Total knee arthroplasty
WOMAC: Western Ontario and McMaster Universities OA index

## References

[1] Turkiewicz A, Gerhardsson de Verdier M, Engstrom G, Nilsson PM, Mellstrom C, Lohmander LS, Englund M (2015). Prevalence of knee pain and knee OA in southern Sweden and the proportion that seeks medical care. Rheumatology (Oxford).

[2] Cooper C, Snow S, McAlindon TE, Kellingray S, Stuart B, Coggon D, Dieppe PA (2000). Risk factors for the incidence and progression of radiographic knee osteoarthritis. Arthritis Rheum.

[3] Silverwood V, Blagojevic-Bucknall M, Jinks C, Jordan JL, Protheroe J, Jordan KP (2015). Current evidence on risk factors for knee osteoarthritis in older adults: a systematic review and meta-analysis. Osteoarthr Cartil.

[4] Weinstein AM, Rome BN, Reichmann WM, Collins JE, Burbine SA, Thornhill TS, Wright J, Katz JN, Losina E (2013). Estimating the burden of total knee replacement in the United States. J Bone Joint Surg Am.

[5] Niu J, Zhang YQ, Torner J, Nevitt M, Lewis CE, Aliabadi P, Sack B, Clancy M, Sharma L, Felson DT (2009). Is obesity a risk factor for progressive radiographic knee osteoarthritis?. Arthritis Rheum.

[6] Bedson J, Croft PR (2008). The discordance between clinical and radiographic knee osteoarthritis: a systematic search and summary of the literature. BMC Musculoskelet Disord.

[7] Chapple CM, Nicholson H, Baxter GD, Abbott JH (2011). Patient characteristics that predict progression of knee osteoarthritis: a systematic review of prognostic studies. Arthritis Care Res (Hoboken).

[8] Riddle DL, Kong X, Jiranek WA (2012). Factors associated with rapid progression to knee arthroplasty: complete analysis of three-year data from the osteoarthritis initiative. Joint Bone Spine.

[9] Raynauld JP, Martel-Pelletier J, Haraoui B, Choquette D, Dorais M, Wildi LM, Abram F, Pelletier JP, Canadian Licofelone Study Group (2011). Risk factors predictive of joint replacement in a 2-year multicentre clinical trial in knee osteoarthritis using MRI: results from over 6 years of observation. Ann Rheum Dis.

[10] Roemer FW, Kwoh CK, Hannon MJ, Hunter DJ, Eckstein F, Wang Z, Boudreau RM, John MR, Nevitt MC, Guermazi A (2015). Can structural joint damage measured with MR imaging be used to predict knee replacement in the following year?. Radiology.

[11] Scher C, Craig J, Nelson F (2008). Bone marrow edema in the knee in osteoarthrosis and association with total knee arthroplasty within a three-year follow-up. Skelet Radiol.

[12] Tanamas SK, Wluka AE, Pelletier JP, Pelletier JM, Abram F, Berry PA, Wang Y, Jones G, Cicuttini FM (2010). Bone marrow lesions in people with knee osteoarthritis predict progression of disease and joint replacement: a longitudinal study. Rheumatology (Oxford).

[13] Felson DT, McLaughlin S, Goggins J, LaValley MP, Gale ME, Totterman S, Li W, Hill C, Gale D (2003). Bone marrow edema and its relation to progression of knee osteoarthritis. Ann Intern Med.

[14] Edwards MH, Parsons C, Bruyere O, Petit Dop F, Chapurlat R, Roemer FW, Guermazi A, Zaim S, Genant H, Reginster JY, Dennison EM, Cooper C, SEKOIA Study Group (2016). High Kellgren-Lawrence grade and bone marrow lesions predict worsening rates of radiographic joint space narrowing; the SEKOIA study. J Rheumatol.

[15] Jorgensen A, Stengaard-Pedersen K, Simonsen O, Pfeiffer-Jensen M, Eriksen C, Bliddal H, Pedersen NW, Bodtker S, Horslev-Petersen K, Snerum LO, Egund N, Frimer-Larsen H (2010). Intra-articular hyaluronan is without clinical effect in knee osteoarthritis: a multicentre, randomised, placebo-controlled, double-blind study of 337 patients followed for 1 year. Ann Rheum Dis.

[16] Altman R, Asch E, Bloch D, Bole G, Borenstein D, Brandt K, Christy W, Cooke TD, Greenwald R, Hochberg M (1986). Development of criteria for the classification and reporting of osteoarthritis. Classification of osteoarthritis of the knee. Diagnostic and therapeutic criteria Committee of the American Rheumatism Association. Arthritis Rheum.

[17] Bellamy N. Western Ontario and McMaster Universities Osteoarthritis Index (WOMAC). 2015. http://www.rheumatology.org/I-Am-A/Rheumatologist/Research/Clinician-Researchers/Western-Ontario-McMaster-Universities-Osteoarthritis-Index-WOMAC. Accessed 08/17 2017.

[18] Skou N, Egund N (2017). Patellar position in weight-bearing radiographs compared with non-weight-bearing: significance for the detection of osteoarthritis. Acta Radiol.

[19] Boegard T, Rudling O, Petersson IF, Sanfridsson J, Saxne T, Svensson B, Jonsson K (1997). Postero-anterior radiogram of the knee in weight-bearing and semiflexion. Comparison with MR imaging. Acta Radiol.

[20] Boegard T, Rudling O, Petersson IF, Sanfridsson J, Saxne T, Svensson B, Jonsson K (1998). Joint-space width in the axial view of the patello-femoral joint. Definitions and comparison with MR imaging. Acta Radiol.

[21] Ahlback S (1968). Osteoarthrosis of the knee. A radiographic investigation. Acta Radiol Diagn (Stockh).

[22] Jørgensen A (2006). Knee osteoarthritis hyaluronan treatment, pain modalities and magnetic resonance imaging: ph.D.-thesis.

[23] Nielsen FK, Egund N, Peters D, Jurik AG (2014). Measurement of bone marrow lesions by MR imaging in knee osteoarthritis using quantitative segmentation methods--a reliability and sensitivity to change analysis. BMC Musculoskelet Disord.

[24] Guermazi A, Roemer FW, Hayashi D, Crema MD, Niu J, Zhang Y, Marra MD, Katur A, Lynch JA, El-Khoury GY, Baker K, Hughes LB, Nevitt MC, Felson DT (2011). Assessment of synovitis with contrast-enhanced MRI using a whole-joint semiquantitative scoring system in people with, or at high risk of, knee osteoarthritis: the MOST study. Ann Rheum Dis.

[25] Rhodes LA, Grainger AJ, Keenan AM, Thomas C, Emery P, Conaghan PG (2005). The validation of simple scoring methods for evaluating compartment-specific synovitis detected by MRI in knee osteoarthritis. Rheumatology.

[26] Danish Health Authority. Knee osteoarthritis - national guidelines. 2012. https://sundhedsstyrelsen.dk/da/udgivelser/2012/~/media/CD7B016D7F9C4766A1530172473FD5F2.ashx. Accessed 01/23 2016.

[27] Danish Health Authority. Knee osteoarthritis, part 2: Visitation rules and hospital treatment. In: Knæartrose. Del 2: Faglige visitationsretningslinjer. 2011. https://www.sundhed.dk/content/cms/34/75734_kn%C3%A6artrose%2D-del-2-faglige-visitationsretningslinjer.pdf. Accessed 11/15 2016.

[28] Oak SR, Ghodadra A, Winalski CS, Miniaci A, Jones MH (2013). Radiographic joint space width is correlated with 4-year clinical outcomes in patients with knee osteoarthritis: data from the osteoarthritis initiative. Osteoarthr Cartil.

[29] Ayral X, Pickering EH, Woodworth TG, Mackillop N, Dougados M (2005). Synovitis: a potential predictive factor of structural progression of medial tibiofemoral knee osteoarthritis -- results of a 1 year longitudinal arthroscopic study in 422 patients. Osteoarthr Cartil.

[30] Felson DT, Chaisson CE, Hill CL, Totterman SM, Gale ME, Skinner KM, Kazis L, Gale DR (2001). The association of bone marrow lesions with pain in knee osteoarthritis. Ann Intern Med.

[31] Barr AJ, Campbell TM, Hopkinson D, Kingsbury SR, Bowes MA, Conaghan PG (2015). A systematic review of the relationship between subchondral bone features, pain and structural pathology in peripheral joint osteoarthritis. Arthritis Res Ther..

[32] Yusuf E, Kortekaas MC, Watt I, Huizinga TW, Kloppenburg M (2011). Do knee abnormalities visualised on MRI explain knee pain in knee osteoarthritis? A systematic review. Ann Rheum Dis.

[33] Felson D (2003). Association of bone marrow changes with worsening of knee osteoarthritis. Ann Intern Med.

[34] Conaghan PG, D'Agostino MA, Le Bars M, Baron G, Schmidely N, Wakefield R, Ravaud P, Grassi W, Martin-Mola E, So A, Backhaus M, Malaise M, Emery P, Dougados M (2010). Clinical and ultrasonographic predictors of joint replacement for knee osteoarthritis: results from a large, 3-year, prospective EULAR study. Ann Rheum Dis.

[35] Bastick AN, Runhaar J, Belo JN, Bierma-Zeinstra SM (2015). Prognostic factors for progression of clinical osteoarthritis of the knee: a systematic review of observational studies. Arthritis Res Ther.

[36] Holla JF, van der Leeden M, Heymans MW, Roorda LD, Bierma-Zeinstra SM, Boers M, Lems WF, Steultjens MP, Dekker J (2014). Three trajectories of activity limitations in early symptomatic knee osteoarthritis: a 5-year follow-up study. Ann Rheum Dis.

