# Supplementary material for: Risk factors for joint replacement in knee osteoarthritis; a 15-year follow-up study
Source: BMC Musculoskelet Disord. 2017 Dec 4;18:510. doi: 10.1186/s12891-017-1871-z (PMC5715644; doi:10.1186/s12891-017-1871-z)
Supplement: Supplementary file 1 — Observer agreement, BML and synovitis grading, Bland-Altman analyses. (DOCX 12 kb) [file 12891_2017_1871_MOESM1_ESM.docx]

| Observer agreements | | | | |
| --- | --- | --- | --- | --- |
| Entity | Observer agreement | Bias | 95% limits of agreement | |
| BML | Intra-observer | 0.30 | 0.08 | 0.53 |
| Synovitis | Intra-observer | -1.4 | -2.3 | -0.5 |
|  | Inter-observer | 0.5 | 0.0 | 1.0 |
| Effusion | Intra-observer | -0.5 | -2.0 | 1.0 |
|  | Inter-observer | 0.9 | -0.3 | 2.0 |

BML = bone marrow lesion
