# Supplementary material for: Risk factors for joint replacement in knee osteoarthritis; a 15-year follow-up study
Source: BMC Musculoskelet Disord. 2017 Dec 4;18:510. doi: 10.1186/s12891-017-1871-z (PMC5715644; doi:10.1186/s12891-017-1871-z)
Supplement: Supplementary file 2 — Observer agreement, BML and synovitis grading, Intraclass correlation coefficients. (DOCX 12 kb) [file 12891_2017_1871_MOESM2_ESM.docx]

| Observer agreements | | | | |
| --- | --- | --- | --- | --- |
| Entity | Observer agreement | ICC | 95% CI | |
| BML | Intra-observer | 0.98 | 0.97 | 0.99 |
| Synovitis | Intra-observer | 0.93 | 0.86 | 0.97 |
|  | Inter-observer | 0.97 | 0.94 | 0.99 |
| Effusion | Intra-observer | 0.89 | 0.76 | 0.95 |
|  | Inter-observer | 0.94 | 0.87 | 0.97 |

ICC: intraclass correlation coefficients; CI = confidence interval; BML = bone marrow lesion
