# Supplementary material for: Risk factors for joint replacement in knee osteoarthritis; a 15-year follow-up study
Source: BMC Musculoskelet Disord. 2017 Dec 4;18:510. doi: 10.1186/s12891-017-1871-z (PMC5715644; doi:10.1186/s12891-017-1871-z)
Supplement: Supplementary file 3 [file 12891_2017_1871_MOESM3_ESM.docx]

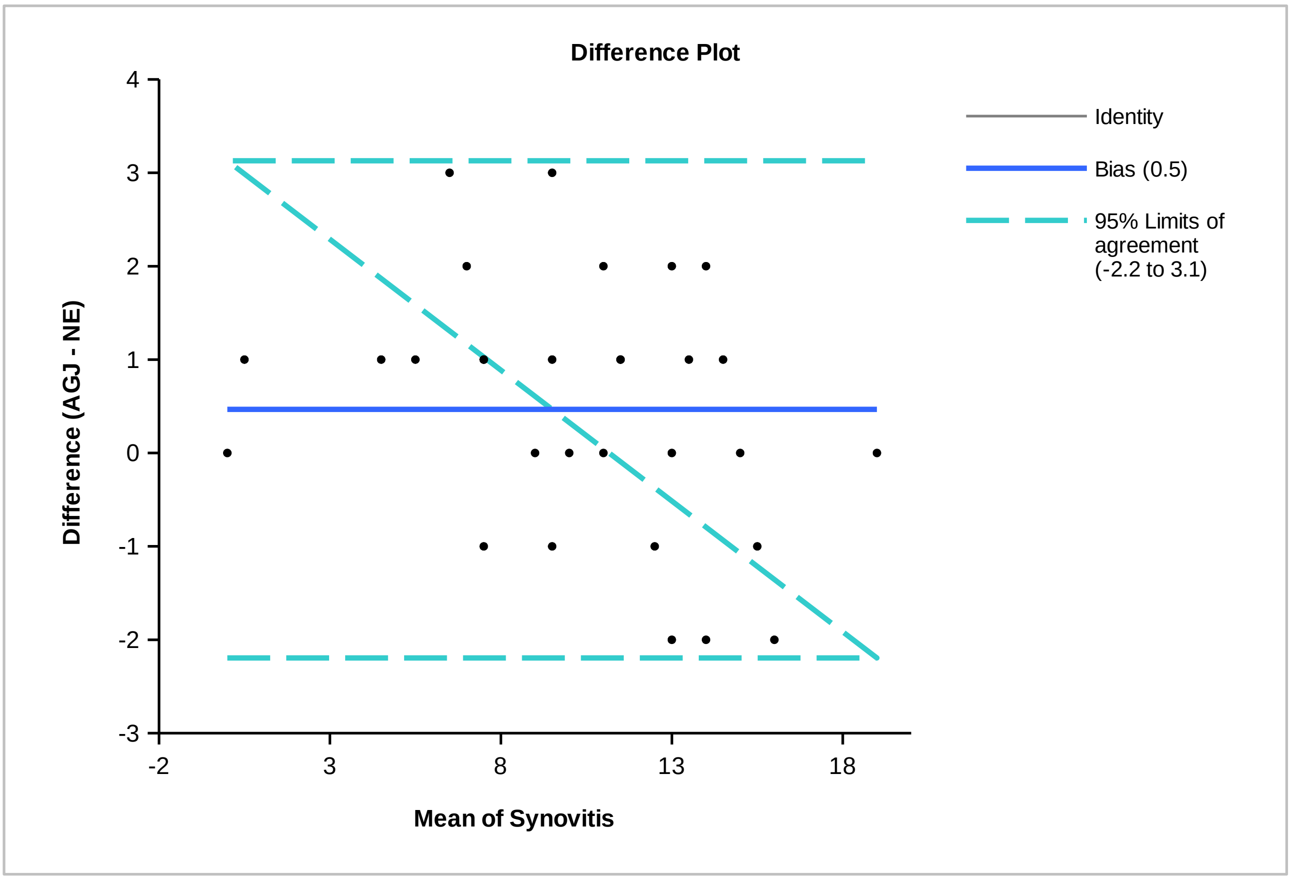

Difference Plot
4
3
2
1
0
-1
-2
-3
Difference (AGJ - NE)
-2
3
8
13
18
Mean of Synovitis
Identity
Bias (0.5)
95% Limits of agreement (-2.2 to 3.1)
