# Supplementary material for: Risk factors for joint replacement in knee osteoarthritis; a 15-year follow-up study
Source: BMC Musculoskelet Disord. 2017 Dec 4;18:510. doi: 10.1186/s12891-017-1871-z (PMC5715644; doi:10.1186/s12891-017-1871-z)
Supplement: Supplementary file 4 [file 12891_2017_1871_MOESM4_ESM.docx]

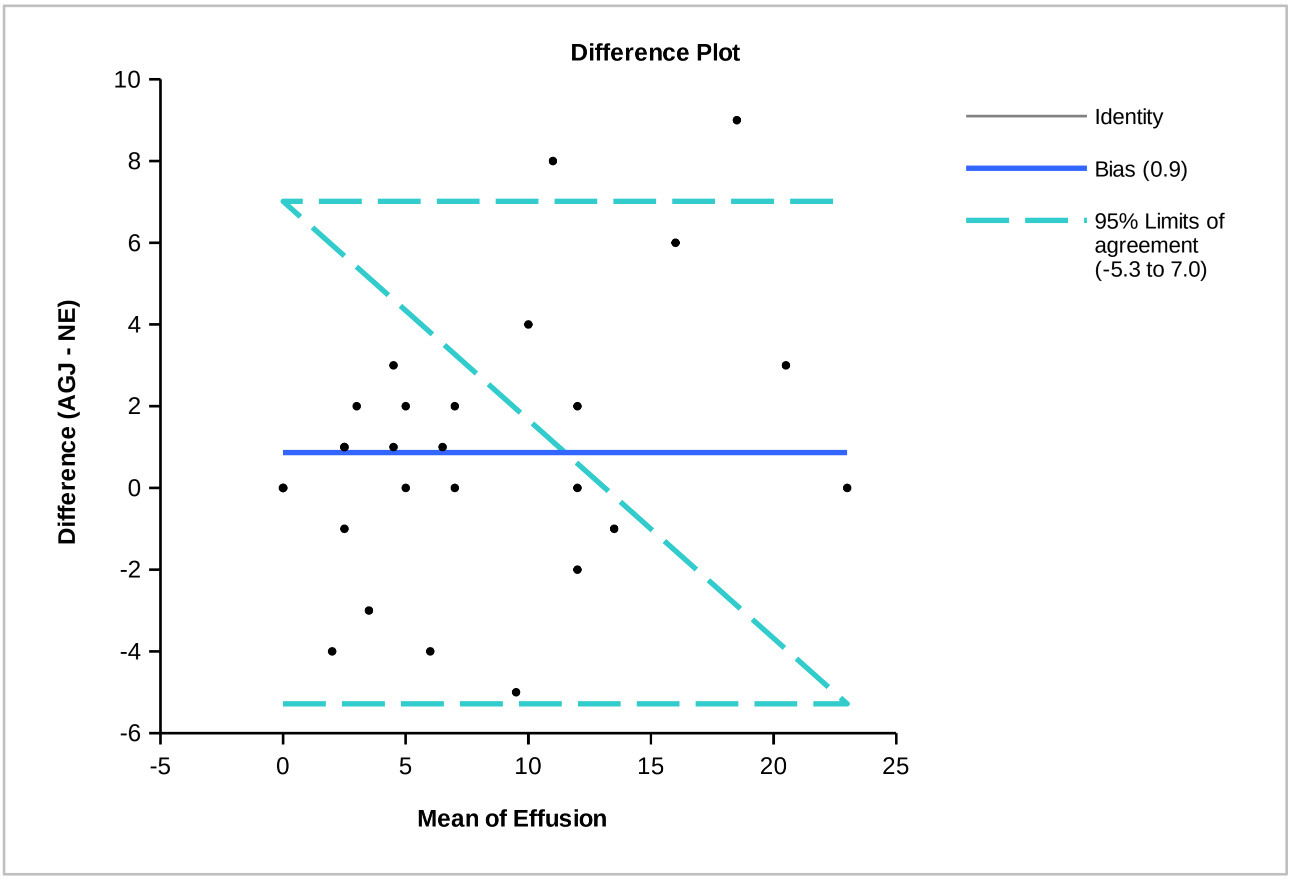
Difference Plot
Difference (AGJ - NE)
Mean of Effusion
10
8
6
4
2
0
-2
-4
-6
-5
0
5
10
15
20
25
Identity
Bias (0.9)
95% Limits of agreement (-5.3 to 7.0)
